# Electroanatomic Contact Mapping: How to Use Optimally to Recognise the Arrhythmia Mechanism?

**Published:** 2010-01-07

**Authors:** Narayanan Namboodiri

**Affiliations:** Sree Chitra Tirunal Institute for Medical Sciences and Technology, Trivandrum, Kerala, India

**Keywords:** arrhythmia, mapping

The evolution in 3-D electroanatomic contact mapping systems has helped to considerably enhance the electrophysiologists' understanding of real-time position management and mapping, thus to enable more precise catheter navigation within heart. These 3-D contact mapping techniques help to define the patient's cardiac anatomy in a better way for successful electrophysiological procedures by taking into account the importance of anatomic structures to the initiation and maintenance of cardiac arrhythmias. A system which plots the position of and activation time at a roving mapping catheter assists in identifying the sites of early activation for focal arrhythmias, and, combined with entrainment mapping, appears to be useful in identifying critical isthmuses in complex reentry circuits. 3D constructs of electrogram voltage may also help define areas of electrical scarring and infarction. Plotted together, these voltage, local activation timing and complex fractionated maps help to demonstrate the complex relation between the anatomical and functional barriers in the genesis of arrhythmias.

 Despite the diversities in the technologies involved, the basic principle of any electroanatomic mapping system combines four essential steps to diagnose arrhythmia: 1) defining chamber anatomy; 2) recording signal morphology, timing, and voltage; 3) displaying the electrical information as color-coded maps, and 4) interpreting these maps to delineate the arrhythmia mechanism, in isolation or combined with other techniques like entrainment mapping. Additional caution in the sequential application of these steps can undoubtedly improve the success of electrophysiological procedure.

## Outlining the 3-D chamber anatomy

The resolution of the map to diagnose an arrhythmia depends on the number of data points taken in any mapping system as higher point numbers reduce anatomical interpolation. However, these systems have limitations when mapping complex and adjoining structures, especially in the areas like the junction between chambers or connections of the chamber with structures like valves, veins or arteries. One way to minimize this 'interpolation obliteration' of those junctions with smoothing over the angles is to treat the adjoining structures as separate volumes or maps, which some systems readily allow. It is a good practice to identify the anatomic structures such as the vena cava, atrioventricular annuli, and pulmonary veins and display them in the initial phase of geometry creation itself. Validating the identity of these anatomical landmarks with fluoroscopy or intracardiac potentials in the initial phase of chamber geometry creation can serve as a skeleton for further mapping and improve its overall diagnostic sensitivity.

## Recording electrical information

Local activation timing requires an anatomically stable and electrically well-defined reference electrogram, to which the activation time of a roving catheter can be compared. The reference electrode should remain stable during the entire study; so confirmation of its position fluoroscopically in relation to other landmarks is essential to ensure its temporal stability. In addition, reproducibility in automatic sensing of the reference is essential for map accuracy. Automated sensing of mapping and reference electrograms is usually accomplished by detecting peak amplitude or maximal slope. Conventionally, maximum amplitude is taken for bipolar electrograms and maximum slope for unipolar electrograms. The steepest negative intrinsicoid deflection in a unipolar electrograms is known to correlate with maximal Na+ conductance [1] and thus, has the advantage of providing precise measurement of local activation times. But poor signal-to-noise ratio, especially in areas of scar or low voltage potentials can compromise the reproducible interpretation of this fiducial point.

While mapping tachycardia, apart from the electrical reference, a window for assigning activation times on the mapping catheter is required.  This is the time interval relative to the fiducial point, during which the local activation time is determined in the mapping (i.e., roving) channel. Only those activation times falling within this window will be acquired. These activation timings are labeled "early" or "late" relative to the electrical reference within this window.  The total length of the window of interest should always be less than the tachycardia cycle length to avoid two activations in the same point falling during this window period. The boundaries of this window relative to the reference electrogram and its range should be set based on the presumed activation range of the tachycardia being mapped. For example, it is important to select a broader range for window, nearly approximating the tachycardia cycle length, while mapping macroreentrant tachycardias. However, while doing so, it is important to remember that the designation of activation time as early or late in a chamber is arbitrary, as some point of the chamber is activated at any given point during macroreentrant atrial flutters. Hence, a change in window of interest in these cases would only result in a phase-shift of the activation pattern, still maintaining the same activation sequence within the circuit.

## Displaying electrical information

**Figure 1:**
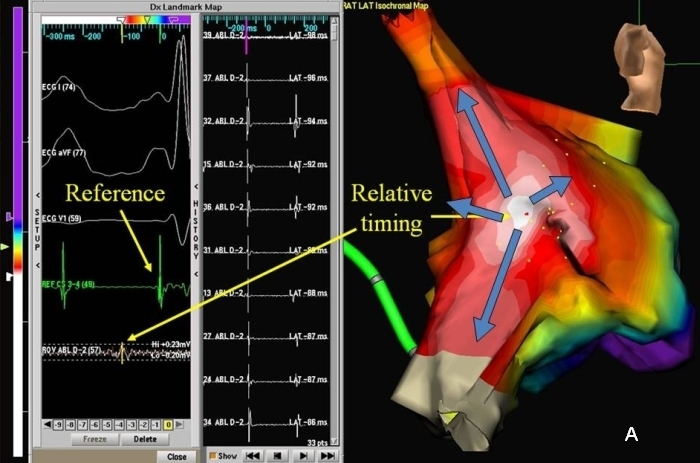

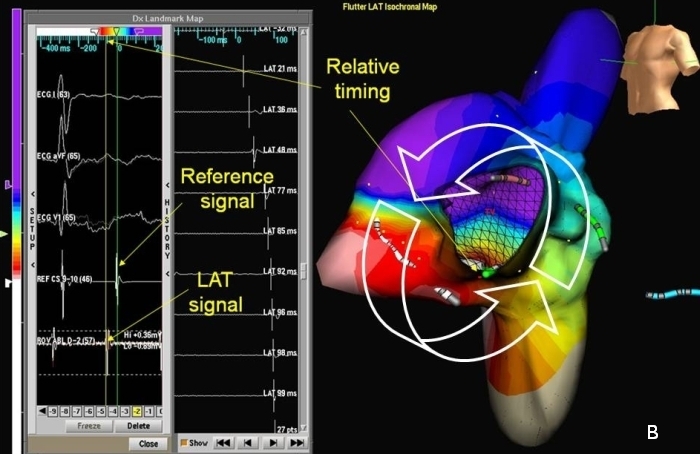
Isochronal mapping of focal (A) and macroreentrant (B) tachycardias. The activation time-scale is color coded with the white followed by red color denoting the earliest sites of activation and the purple the last activated site. Figure A shows the centrifugal activation from the mid-crista of right atrium in a patient with atrial tachycardia of focal mechanism. Only 116msec could be mapped in the right atrium while the tachycardia cycle length was 286ms. Figure B shows mapping of right atrial macroreentrant tachycardia. The tachycardia cycle length was 236ms. 220ms (>90%) of the flutter cycle length could be mapped in the right atrium.  The wave-front propagation pattern, which is perpendicular to the isochrones, suggests typical flutter in this case.

Electrical information obtained during the signal recording can be presented in the form of activation timing, voltage amplitude, or other user-defined measurements. The values obtained are color-coded and assigned to the positional location of the roving catheter. These values can be used to generate activation, isochronal, propagation, voltage or signal complexity maps depending upon the nature of arrhythmias. Activation maps display color-coded local activation time superimposed on the chamber geometry, and are useful for mapping both focal and macroreentrant tachycardias (Figure 1) and to demonstrate 'activation detour' following linear ablation. The propagation maps show the dynamic propagation of active wave-front across the endocardial chambers. As per conventional color-coding for activation and propagation mapping, red indicates the early activation sites, blue and purple late activated sites, and yellow and green the intermediate local activation sites. Voltage maps are created by the system based on the peak-to-peak amplitude of the local electrograms. The value is color-coded with purple and red representing areas of highest and lowest amplitude respectively. Conventionally, bipolar voltage amplitude <1.5 mV is considered abnormal in the ventricles, and an electrically "dense scar" is inferred in regions demonstrating voltages < 0.5 mV [2]. However, in the atria, scar is defined as an area with bipolar voltage less than 0.05 mV [3-5]. This approach is highly useful to identify the areas of electrical scarring and defining the potential channels within it. However, one should remember that these areas with low voltage ('electrical scarring') do not necessarily represent areas of pathological scar. Signal complexity maps, another way of depicting the electrical information, are often generated based on user-defined algorithms.

The accuracy of electroanatomic maps largely depends on their spatial resolution and temporal stability. High density maps are often required near the area of interest to avoid erroneous interpolation between distant neighboring points and thus misinterpretation of the map. Similarly, identification and annotating the presence and location of double potentials, fractionated electrograms and pathway, diastolic or Purkinje potentials on the anatomic shell may superiorly define the tachycardia circuit and may even provide additional diagnostic information for targeted ablation.

## Interpretation of the maps

The primary aim of the electroanatomic maps is to assist in defining the tachycardia mechanism and thereby its treatment. A focal tachycardia is characterized by a localized area of early activation where the electrical signals usually precede the surface P or QRS by 30-70ms (Figure 1A). From this point of early activation, the impulse spreads in a centrifugal fashion, which can be depicted by the color-coded activation or propagation maps [6]. In a focal mechanism, the points of the earliest and the latest points of electrical activation are anatomically well separated in the chamber being mapped, unless a unidirectional conduction pattern results close to the focus of arrhythmia due to fixed or functional conduction block. Although regions of conduction block and slow conduction are not unusual bystanders in a focal mechanism, they are not critical for maintenance of the tachycardia circuit. The total chamber activation time in these tachycardias represent the conduction time from the earliest to the last point of activation time within the chamber, and is expected to be substantially less than the tachycardia cycle length.

In contrast, macroreentrant tachyarrhythmias would show adjacent areas of early and late activation, separated by areas of intermediate values of activation (Figure 1B). More than 90% of the tachycardia cycle length can be mapped in macroreentrant tachycardias. Another useful feature of the mapping software helps to detect 'early meets late activation' pattern. By coding these adjacent regions a specific color, sequential activation of the late to early sites can be depicted, as would be expected in a reentrant circuit. This algorithm avoids interpolation of activation times between the early and late points and avoids the false impression of a focal mechanism to the tachycardia.  The direction of propagation of the reentrant wave-front can be interpreted based on the isochronal maps, which runs perpendicular to the isochronal lines. However, the radial activation of the bystander sites can confound interpretation of isochronal maps in many cases. It is advisable to combine entrainment mapping with electroanatomic mapping in these situations.

Increasingly, the role of localized reentry in the genesis of arrhythmias is recognized, particularly occurring in patients with previous surgery or ablation. Unfortunately, unless the electrograms are carefully scrutinized these arrhythmias may be missed despite the application of these mapping systems. Thus, it is important to combine the findings of conventional mapping and entrainment techniques with that obtained from the 3D mapping systems for expeditious and accurate mapping of arrhythmias and interpretation of its mechanism.

## Possible caveats in this sequential approach

Although electroanatomic mapping systems have largely contributed to better understanding of the electrophysiologic substrate predisposing to various clinical arrhythmias [7,8] and thereby improvements in the approach to complex arrhythmias [9-19], the correct interpretation of the electrical information obtained during mapping is still underpinned by basic electrophysiology principles.  The following sources of error need to be considered during this process:

1. *Areas of anatomical barriers and fixed conduction block.* Many areas in the heart like crista terminalis, scars, veno-atrial junctions, valves or even infarct-related scars can act as areas of conduction block. The interpolation of activation through these areas of conduction block can give an appearance of focal arrhythmia with centrifugal spread of activation, masking a macroreentrant mechanism. Dense mapping in these areas and looking for the presence of double potentials can often help in discrimination.
2. *Low resolution maps.* A poor-resolution map can lead on to incorrect interpretation about components of the arrhythmia circuit. Interpolation between anatomically distant activation times may hide the true mechanism of arrhythmia. Radial activation from a macroreentrant circuit can also give intermediate activation times in the surrounding region not directly involved in the circuit. The direction of wave-front propagation can be correctly interpreted in these cases by the 'early meets late' algorithm and entrainment mapping. Similarly, dense mapping is often required in the scar region in cases of scar-related atrial or ventricular complex reentrant arrhythmias to identify the low voltage signals, which may serve as potential targets of ablation in the diastolic isthmus [10].
3. *Identification of the chamber(s) harboring arrhythmia.* Arrhythmias arising and limited to one of the chambers can be commonly mistaken for that arising from the other. This is especially true for focal arrhythmias arising close to septum. Furthermore, if mapping is limited to the right atrium, left atrial tachycardias may be mistaken for focal arrhythmias originating in the region of dominant interatrial conduction like Bachmann bundle, the interatrial septum, or the coronary sinus. Similarly, ventricular tachycardias arising from left ventricular outflow including aortic sinuses may be mistaken for arrhythmias arising from right ventricular outflow region if only right ventricle is mapped. Broader areas of early activation along the intervening septum, and the absence of significant prematurity of the local activation time in the mapped areas in relation to the P wave or QRS can serve as a clue in recognizing this septal breakthrough. In addition, the utility of entrainment mapping from the septal regions in the identification of these septal breakthroughs during macroreentrant tachycardias cannot be overemphasized.
4. *Inappropriate activation window.* Focal arrhythmias with adjacent areas of conduction blocks or slow passive activation of the chamber with prolonged chamber activation time are not unusual. If the activation window selected approximates the tachycardia cycle length, this could result in ambiguous map especially in tachyarrhythmias with cycle length variations. This inappropriate window selection may generate a spurious "early meets late activation" pattern mimicking a macroreentrant mechanism. Additional measures like identification of the functional and anatomical barriers contributing to this delay and use of entrainment mapping can be useful in these settings.
5. *Complex reentrant circuits.* The components of circuits in reentrant arrhythmias such as double loop reentrant tachycardias may remain undetected unless suspected and specifically looked for. A low resolution map can particularly contribute to this problem. Dense mapping, noting the markers of local conduction abnormalities like the fractionated electrogram, double potentials or local conduction delay, and entrainment mapping can help to identify and localize the reentrant circuits in these cases. Similarly, in scar-related ventricular tachycardias, maps solely based on the activation timing and voltage of the signals should be interpreted with caution. As in any reentrant circuits, many innocent bystander loops can have similar activation timings without necessarily being part of the circuit. Recognition of diastolic isthmus in these complex reentrant circuits may demand supplementary entrainment maneuvers.
6. *Fractionated electrograms.* Assigning an activation time for highly fractionated and wide potentials requires a cautious approach. In bipolar maps, larger amplitude signals usually represent the local activation time; mis-annotation of these points may lead to incorrect map interpretation.
7. *Movement artifacts.* Movement artifacts occurring after the geometry has been acquired can distort the anatomical correlation. Cardiac and respiratory movements may decrease the accuracy of catheter localization.  If the mapping has been performed during various activation sequences such as sinus rhythm, arrhythmia or cardiac pacing, apparent shifts in the catheter localization might occur because of change in chamber geometry and gating.  An intracardiac spatial reference will largely reduce the shifts due to the respiratory excursions.

It is important to remember that the use of these advanced mapping systems, capable of three-dimensional rendering of cardiac chambers and superimposition of electrical information, are not designed to replace conventional mapping techniques but to be used as an adjunctive tool in the analysis and treatment of complex arrhythmias.
